# COVID-19 and the eye: how much do we really know? A best evidence review

**DOI:** 10.5935/0004-2749.20200067

**Published:** 2020

**Authors:** Juan Pablo Olivares-de Emparan, Carolina Sardi-Correa, Juan Alberto López-Ulloa, Jaime Soria-Viteri, Jason A. Penniecook, Jesús Jimenez-Román, Van C. Lansingh

**Affiliations:** 1 Asociación para evitar la ceguera en México, Mexico City, Mexico; 2 Centro Mexicano de Salud Visual Preventiva, Mexico City, Mexico; 3 Instituto Nacional de Investigación en Oftalmología, Medellin, Colombia; 4 Universidad de Especialidades Espíritu Santo, Guayaquil, Ecuador; 5 Universidad Católica Santiago de Guayaquil, Guayaquil, Ecuador; 6 Instituto de la Visión, Montemorelos University, Montemorelos, Mexico; 7 Instituto Mexicano de Oftalmología, Queretaro, Mexico; 8 Help Me See, New York City, NY, USA

**Keywords:** COVID-19, Ophthalmology, SARS-CoV-2, Conjunctiva, Ocular, COVID-19, Oftalmologia, SARS-CoV-2, Conjuntiva, Ocular

## Abstract

To identify and classify available information regarding COVID-19 and eye care according to the level of evidence, within four main topics of interest: evidence of the virus in tears and the ocular surface, infection via the conjunctival route, ocular manifestations, and best practice recommendations. A structured review was conducted in PubMed, ScienceDirect, LILACS, SciELO, the Cochrane Library and Google Scholar on COVID-19 and ophthalmology. The Oxford Centre for Evidence Based Medicine 2011 Levels of Evidence worksheet was used for quality assessments. 1018 items were identified in the search; 26 records were included in the qualitative synthesis, which encompassed 6 literature reviews, 10 case series or cross-sectional studies, 4 case reports, and 6 intervention descriptions. Seventeen out of 26 records (65%) were categorized as level 5 within the Oxford CBME methodology grading system, the rest were level 4. The evidence generated on COVID-19 and ophthalmology to date is limited, although this is understandable given the circumstances. Both the possible presence of viral particles in tears and conjunctiva, and the potential for conjunctival transmission remain controversial. Ocular manifestations are not frequent and could resemble viral infection of the ocular surface. Most recommendations are based on the strategies implemented by Asian countries during previous coronavirus outbreaks. There is a need for substantive studies evaluating these strategies in the setting of SARS-CoV-2. In the meantime, plans for applying these measures must be implemented with caution, taking into account the context of each individual country, and undergo regular evaluation.

## Abstract (pt)

Identificar e classificar as informações disponíveis sobre o COVID-19 e o tratamento oftalmológico de acordo com o nível de evidência, dentro de quatro tópicos principais de interesse: evidência do vírus nas lágrimas e na superfície ocular, infecção pela via conjuntival, manifestações oculares e recomendações de melhores práticas. Foi realizada uma revisão estruturada no PubMed, ScienceDirect, LILACS, SciELO, Biblioteca Cochrane e Google Scholar no COVID-19 e oftalmologia. A planilha de Níveis de Evidência 2011 do Oxford Centre for Evidence Based Medicine 2011 foi usada para avaliações de qualidade. Mil e dezoito itens foram identificados na busca; Foram incluídos 26 registros na síntese qualitativa, que incluiu 6 revisões de literatura, 10 séries de casos ou estudos transversais, 4 relatos de casos e 6 descrições de intervenções. Dezessete dos 26 registros (65%) foram classificados como nível 5 no sistema de classificação da metodologia Oxford CBME, o restante foi no nível 4. As evidências geradas no COVID-19 e na oftalmologia até o momento são limitadas, embora isso seja compreensível dadas as circunstâncias. Tanto a possível presença de partículas virais em lágrimas e conjuntiva quanto o potencial de transmissão conjuntival permanecem controversos. As manifestações oculares não são frequentes e podem se assemelhar a infecção viral da superfície ocular. A maioria das recomendações baseia-se nas estratégias implementadas pelos países asiáticos durante surtos anteriores de coronavírus. Há necessidade de estudos aprofundados avaliando essas estratégias no cenário da SARS-CoV-2. Enquanto isso, os planos para a aplicação dessas medidas devem ser implementados com cautela, levando em consideração o contexto de cada país e submetidos a auditorias periódicas.

## INTRODUCTION

Coronavirus disease 19 (COVID-19) is caused by the Severe Acute Respiratory Syndrome coronavirus type 2 (SARS-CoV-2), previously named 2019 novel coronavirus (2019-nCoV)^(1)^. The ongoing SARS-CoV-2 pandemic has been linked to ophthalmology since its beginnings. On December 30, 2019, Dr. Li Wenliang, a Chinese ophthalmologist, warned his colleagues through the social network WeChat about a SARS-like outbreak in Wuhan and its possible link to a local market^(2)^. On January 30, 2020, the World Health Organization (WHO) confirmed the outbreak as a public health emergency of international interest^(3)^, and on March 11, 2020 it was declared a pandemic^(4)^.

Coronaviruses (CoVs) are known to cause infections in humans and other mammals^(5,6)^, they gained public attention during the Severe Acute Respiratory Syndrome (SARS) outbreak in East Asia in 2003 and the spread of Middle Eastern Respiratory Syndrome (MERS) in 2012^(5,7)^. SARS-CoV-2 is a single-stranded, positive-sense, enveloped RNA virus^(1)^. The main method of transmission is human-to-human through direct contact and droplets; transmission from asymptomatic carriers has also been reported^(8)^. Viral stability in aerosols on different surfaces has been demonstrated^(9)^, supporting evidence on indirect viral acquisition from fomites^(10)^, through the mucous membranes of the mouth, nose and eyes^(11)^. At present, conjunctival transmission of CoVs has not been confirmed and remains controversial.

Out of the seven types of human coronaviruses (HCoV), HCoV-NL63 is the only one that has been confirmed to produce ocular disease, specifically conjunctivitis^(12)^, although the pathogenic mechanism of ocular infection has not been elucidated^(2)^. Ocular symptoms from SARS-CoV and MERS-CoV, which produce similar respiratory manifestations to SARS-CoV-2, have not been reported^(2)^. On the other hand, detection of SARS-CoV RNA in tears was confirmed in three out of 36 patients with SARS during the 2003 outbreak; further studies failed to confirm these results^(13,14)^.

Reports on SARS-CoV-2 conjunctival transmission, viral shedding through tears, and ocular manifestations in COVID-19 patients have emerged^(15-17)^. As the current pandemic progresses, so does the need for reliable information to help generate evidence-based recommendations on best practices. Incentives to rapidly produce scientific reports and positive results lead to the risk of disseminating poorly supported evidence and overloading the public with uncertain or even false information^(18,19)^. In this review we aim to identify the available information regarding COVID-19 and eye care and to classify it according to four main topics of interest: evidence of the virus in tears and the ocular surface, infection via the conjunctival route, ocular manifestations, and best practice recommendations.

## METHODS

### Search strategy and searching other sources

A search strategy was developed using MeSH terms and free-text terms in the following databases: PubMed, ScienceDirect, LILACS, Scielo and the Cochrane Library. A verification search was performed on Google Scholar to identify articles on archival services such as bioRxiv, medRxiv and others.

The search strategy was designed to identify studies providing data on issues related to COVID-19 and ophthalmology. The final search was conducted on April 21. Table 1 lists the keywords included in the search strategy, which were as follows: (“ophthal*” OR “ocular” OR “vision” OR “visual” OR “eye” OR “conjunctiv*” OR “tear”) AND (“covid-19” OR “covid19” OR “2019-nCoV” OR “coronavirus” OR “coronavirus19” OR “coronavirus-19” OR “SARS-Cov-2” OR “severe acute respiratory syndrome 2” OR “SARS2”). Finally, we searched the reference lists of the identified screened publications.

**Table 1:** List of keywords used for the search strategy

COVID-19	Ophthalmology
COVID19	Ophthalmic
2019-nCoV	Ocular
Coronavirus	Visions
Coronavirus-19	Visual
Coronavirus19	Eye
SARS-Cov-2	Conjunctiva
Severe acute respiratory syndrome coronavirus 2	Conjunctivitis
	Tear

### Study selection and data extraction

Results were limited to publications from 2020, as the report from Chinese authorities to the WHO was filed on December 31, 2019. No language restrictions were used, and all publication types were retrieved. Articles in languages other than English, Spanish, Portuguese and French were translated using three different online platforms: Google Translator^®^, DocTranslator^®^ and DeepL^®^; all included articles had at least an abstract available in English. Duplicates were excluded.

Two reviewers independently extracted data using a pre-defined template (Microsoft^®^ Excel spreadsheet). Disagreements were resolved through discussion or by a third reviewer. All articles that required an online platform translation were assessed by three reviewers.

### Data synthesis

Previous validated methods were selected to assess the appropriateness of the publications: CARE^(20)^ guidelines checklists for case reports and case series, and STROBE^(21)^ checklists for observational studies. A narrative approach was used to synthesize the extracted data. The Oxford Centre for Evidence Based Medicine (OCEBM) 2011 Levels of Evidence worksheet was used for quality assessments.^22^ The risk-of-bias assessment used a qualitative approach, taking into consideration the study design, limitations in the methodology and the rigor of execution.

During the selection phase, it was noted that the identified articles corresponded to a limited number of authors. A reference list was created to determine the number of citations for each author when reporting on this topic.

Ethics approval was not required, as the review involved publicly available data. The report was conducted in accordance with the requirements of the Preferred Information Elements for Systematic Testing and Me ta-Analyses (PRISMA)^(23)^.

## RESULTS

### Search results

**Figure 1:**
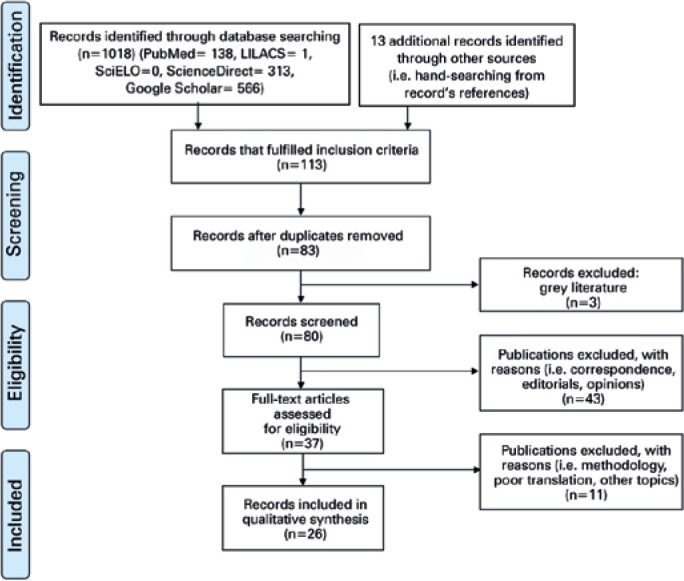
Methodology flowchart. Adapted from the Preferred Information Elements for Systematic Testing and Meta-Analyses (PRISMA)^23^.

A total of 1018 records were retrieved from databases and ten others were found through referencing. After screening by title and/or abstract, 918 records were excluded. Duplicates and grey literature were removed, leaving 80 records which met the eligibility criteria. Correspondence and editorials accounted for 43 records (54%) which were reassessed and excluded. A full text assessment for eligibility was carried out for the remaining documents. Further analysis led to the exclusion of three publications which were deemed opinion pieces, four due to incoherent translation; and three others, which focused on topics outside the scope of this review. One case report was excluded as it did not fulfill the CARE checklist guidelines. Finally, 26 records were included in the qualitative synthesis: 6 literature reviews, 10 case series or cross-sectional studies, 4 case reports, and 6 intervention descriptions (miscellaneous) (Figure 1).

### Included studies

The included studies are presented in tabular form in Table 2. Additional information is provided regarding the author, journal, type of study, main question and other comments. Seventeen out of 26 records (65%) were categorized as level 5 within the Oxford CBME methodology grading system, the rest were level 4.

**Table 2:** Records included in the qualitative analysis: main characteristics and Oxford Center for Evidence Based Medicine (OCEBM) score

Authors and reference	Study type	Main question	Peer reviewed	OCEBM methodology grading
Seah I, et al.^(6)^	Literature review	What is the evidence on ocular compromise by coronaviruses?	Yes	5
Yu A, et al.^(24^	Literature review	What do we know so far about SARS-CoV-2 and ocular manifestations?	Yes	5
Romano M, et al.^(25)^	Literature review	What preventive strategies can personnel in an ophthalmology department employ to reduce transmission?	Yes	5
Jones L, et al.^(26)^	Literature review	Are there special measures that contact-lens wearers should consider?	Yes	5
Sadhu S, et al.^(27)^	Literature review	What measures could be implemented in an ophthalmic facility?	Yes	5
Sarma P, et al.^(28)^	Literature review and meta-analysis	What is the evidence of ocular manifestations and PCR positivity in COVID-19 patients?	No; pre-print	4
Yu Jun I, et al.^(29^	Cross sectional study	Are there viral particles on the ocular surfaces of COVID-19 patients?	Yes; journal pre-proof	4
Guan WJ, et al.^(30)^	Cross sectional study	What are the clinical manifestations of COVID-19 patients according to severity of disease?	Yes	4
Deng C, et al.^(31)^	Cross sectional study	Are there viral particles in conjunctiva or tears in COVID-19 pneumonia patients?	No; pre-print	4
Zhang X, et al.^(32)^	Cross sectional study	Are there viral particles on the ocular surfaces of COVID-19 patients with ocular manifestations?	Yes; journal pre-proof	4
Chen L, et al.^(33)^	Cross sectional study	What are the ocular manifestations of COVID-19 patients?	No; pre-print	4
Xia J, et al.^(15)^	Case series	Are there viral particles on the ocular surfaces of COVID-19 patients?	Yes	4
Wu P, et al.^(16)^	Case series	What are the ocular findings in COVID-19 patients?	Yes	4
Zhou Y, et al.^(34)^	Case series	Is there evidence to suspect interpersonal transmission of SARS-CoV-2 via aerosol contact with the conjunctiva?	No; pre-print	4
Lan Q, et al.^(35)^	Case series	What are the ocular findings on slit lamp exam in COVID-19 patients?	Yes	5
Zhou Y, et al.^(36)^	Case series	Are there viral particles on the ocular surfaces of COVID-19 patients with ocular manifestations?	Yes; journal pre-proof	5
Colavita F, et al.^(37)^	Case report	N/A	Yes	5
Chen Lu, et al.^(38)^	Case report	N/A	Yes	5
Cheema M, et al.^(39)^	Case report	N/A	Yes	5
Li XJ, et al.^(40)^	Case report	N/A	Yes	5
Lai THT, et al.^(41)^	Miscellaneous (Intervention)	What preventive measures can be implemented to reduce the risk of transmission?	Yes	5
Wang N, et al.^(42)^	Miscellaneous (Intervention)	What preventive strategies can personnel take to reduce transmission?	Yes	5
Zhang MC, et al.^(43)^	Miscellaneous (Intervention)	What measures for ophthalmic equipment and personnel can reduce transmission?	Yes	5
Society of Public Health Ophthalmology, Chinese Preventive Medicine Association^(44)^.	Miscellaneous (Intervention)	What measures can personnel take to reduce transmission?	Yes	5
Sengupta S, et al.^(45)^	Miscellaneous (Intervention)	What measures could be implemented in an ophthalmic practice setting?	Yes	5
Gharebaghi R, et al.^(46)^	Miscellaneous (Intervention)	What preventive strategies can personnel take in an ophthalmology department to reduce transmission?	Yes	5

N/A= Not applicable.

The six reviews focused on different subjects: 1) ocular involvement of coronaviruses in humans and animals^(6)^, 2) variations of ocular manifestations^(24)^, 3) preventive strategies in hospital-based ophthalmology departments^(25)^, 4) recommendations for contact lens practices^(26)^, 5) recommendations for eye care facilities^(27)^, and 6) evidence of ocular manifestations and PCR positivity in COVID-19 patients^(28)^. The ten case-series or cross-sectional studies reported findings regarding ocular manifestations; six reported on conjunctival and tear samples tested for viral material. Nine studies were carried out in China, and one in Singapore. Four case reports provide detailed descriptions of ocular manifestations in COVID-19 patients. The six intervention descriptions present recommendations for ophthalmology departments and healthcare workers (HCW) to decrease the risk of transmission. Three of these were extracted from Chinese literature and include recommendations by the Society of Public Health Ophthalmology, Chinese Preventive Medicine Association. The other three papers were from Indian, Iranian and Italian authorship. The publication from India included recommendations from the All India Ophthalmological Society.

### Risk of bias

Due to the purely descriptive nature of the publications and their limited internal validity, they pose a high risk of bias. Qualitative assessment determined the presence of various confounders and covariates that may have influenced the results, and consequently the final analyses.

### Primary outcomes

#### Evidence of viral particles on the ocular surface

Eight case series or cross-sectional studies and four case reports included testing for the presence of viral particles or genetic material from SARS-CoV-2. Ten studies collected samples using conjunctival swabs and performed reverse-transcription polymerase chain reaction (RT-PCR) assay for the detection of viral RNA. One cross-sectional study used tears collected via Schirmer strip and performed viral isolation through Vero-6 cell inoculation. Another study used conjunctival swabs for RT-PCR and Vero-6 cell inoculation. The four case reports observed positive results for RT-PCR of conjunctival swabs; one of them reports on a patient with repetitive positive conjunctival swabs from day 3 to 21 with samples taken almost daily. The results are summarized in table 3.

**Table 3:** Studies reporting evidence of viral particles on the ocular surface

Authors	Collection method/type of test	Tested positive	Percentage positive	Day of sampling	Remarks
Yu Jun 1, et al.^(29)^	Schirmer strips/viral isolation with cell culture and qRT-PCR	0/17	Null	Between days 3 and 20	- Samples collected over a 3-week span (64 samples). - The number of conjunctival swabs varied among patients.
Deng C, et al.^(31)^	Conjunctival swabs / qRT-PCR	0/114	Null	11 ± 6.3	
Zhang X, et al.^(32)^	Conjunctival swabs / RT-PCR	1/72 (confirmed)^*^ 1/102 (total)	1.4% (confirmed)^*^ 0.9% (total)	18.15 ± 7.57	
Xia J, et al.^(15)^	Conjunctival swabs / RT-PCR	1/30	3.33%	7.33 ± 3.82	- Two samples per patient, at 2- to 3-day intervals. - The only patient with ocular manifestations (conjunctivitis) tested positive in 2 samples.
Wu P, et al.^(16)^	Conjunctival swabs/RT-PCR	2/28 (confirmed)^*^ 2/38 (total)	7.14% (confirmed)^*^ 5.26% (total)	Not specified	
Zhou Y, et al.^(34)^	Conjunctival swabs/qRT-PCR	1/63 (confirmed)^*^ 1/67 (total)	1.6% (confirmed)^*^ 1.4% (total)	Not specified	- Two patients (2.9%) had probable positive results.^Λ^ - One patient with conjunctivitis had a negative result in conjunctiva but positive NP.
Lan Q, et al.^(35)^	Conjunctival swabs/RT-PCR	0/81	Null	Not specified	- Examinations happened days after the initial ocular complaints
Zhou Y, et al.^(36)^	Conjunctival swabs/qRT-PCR	3/121	2.5%	Not specified	
Colavita F, et al.^(37)^	Conjunctival swabs/qRT-PCR, viral isolation with cell culture	1	100% (Case report)	Day 3 to 21, almost daily	Case report Cytopathic effects were observed in Vero-6 cells; viral replication was confirmed via qRT-PCT
Chen Lu, et al.^(38)^	Conjunctival swabs/qRT-PCR	1	100% (Case report)	14	Case report
Cheema M, etal.^(39)^	Conjunctival swabs/qRT-PCR	1	100% (Case report)	6	Case report
Li XJ, et al.^(40)^	Conjunctival swabs/RT-PCR	2	100% (Case report)	2	Case report
Sarma P, et al.^(28)^	Conjunctival swabs/RT-PCR or qRT-PCR	(5 studies, 320 patients)	1.95% (95% C.I. 0.74 to 4.11)	Pooled analysis	Pooled analysis

NP= nasopharyngeal swab; RT-PCR= reverse transcription polymerase chain reaction; qRT-PCR= real-time qualitative reverse transcription polymerase chain reaction.

* Study makes a distinction between COVID-19 patients confirmed via nasopharyngeal swabs and those who presented only clinical symptoms. Percentage is shown both for laboratory-confirmed and for the total number of patients.

^Probable positive result definition is not stated in the manuscript.

In all these studies, the percentage of patients with evidence of viral particles in tears or conjunctiva remained low, ranging from 0 to 7.14%. Across studies, there were variations in testing procedures and the type of patients tested. The days at which the samples were taken also show significant variation. The earliest timing was reported as early as day 2 after the initial onset of symptoms. Four studies do not specify the timing of the samples. Case reports included a total of five cases with ocular manifestations; all of these tested positive via conjunctival RT-PCR.

In their review, Sarma et al.^(28)^ conducted a meta-analysis of pooled patients across 5 studies (four items included in the current revision; the other one was discarded during the screening process). They concluded that the proportion of conjunctival/tear sample that were positive for the virus was 1.95% (95% C.I. 0.74 to 4.11).

The literature review by Seah et al. comments on the similarities of SARS-CoV-2 to other CoVs with the possibility of viral shedding in conjunctiva and tears^(6)^. Yu A et al. mention the proven evidence of aerosol formation through air puff tonometry and the possibility of transmission through contact with conjunctiva^(24)^. They both conclude that the evidence about the virus in tears and on the ocular surface remains controversial.

#### The conjunctiva as an infection route

A few anecdotal reports describe non-specific ocular symptoms as the first manifestation of COVID-19. We found one case report of keratoconjunctivitis as the initial presentation. Additionally, Li et al. describe two cases of HCW who tested positive for SARS-CoV-2 in nasopharyngeal (NP) and conjunctival swabs, despite having worn appropriate personal protective equipment (PPE) except for eye protection^(40)^. The first case involved an anesthesiologist who performed an intubation procedure on a COVID-19 patient without ocular protection. She later presented with red eye and viscous conjunctival discharge; after three days she developed respiratory symptoms and was diagnosed with COVID-19. The second case was a nurse with respiratory symptoms and pruritus as well as conjunctival congestion. Both reports support the theory of the conjunctival mucosa acting as an entrance route for the virus.

#### Ocular manifestations

The ten case series or cross-sectional studies reported ocular manifestations, particularly conjunctival congestion. Four case reports presented with signs compatible with follicular conjunctivitis and one as unilateral keratoconjunctivitis. The timing of onset of ocular manifestations varies across the studies, with these symptoms appearing to be most prominent in the early stages of the disease. Most manifestations are bilateral and seem to cause little to no discomfort. Only the study by Chen et al. focuses on patient-reported ocular symptoms^(33)^, while the remaining case series focus on observable signs. The results are summarized in table 4.

**Table 4:** Reported ocular manifestations in COVID-19 patients

Author	Patients with ocular manifestations (percentage)	Ocular manifestations	Onset of manifestations	Ocular manifestations collection methods
Yu Jun 1, et al.^(29)^	0/17	Null	-	EMR
Guan WJ, et al.^(30)^	9/1009 (0.9%)	- Conjunctival congestion	Not specified	EMR
Deng C, et al.^(31)^	0/114	Null	-	EMR
Zhang X, et al.^(32)^	2/72 (2.8%)^*^ 2/102 (1.96%)	- Epiphora - Conjunctival hyperemia - Normal visual acuity	Day 2 to 5	Not specified
Chen L, et al.^(33)^	25/534 (4.7%)	- Conjunctival congestion - Secretion - Dry eye (20.97%) - Blurred vision (12.73%) - Foreign body sensation (11.80%) - Ophthalmalgia - Photophobia	Day 8 to 12	Ophthalmologists via telephone, face-to-face survey, or smartphone application
Xia J, et al.^(15)^	1/30 (3.3%)	- Conjunctival hyperemia - Aqueous secretion	Day 3	Not specified
Wu P, et al.^(16)^	12/38 (31.6%)	- Chemosis - Epiphora - Conjunctival hyperemia - Secretion	Not specified	Not specified
Zhou Y, et al.^(34)^	1/63 (1.6%)^*^ 1/67(1.5%)	- Conjunctival hyperemia - Aqueous secretion	Not specified	Retrospectively patient-reported outcomes (survey)
Lan Q, et al.^(35)^	3/81 (3.7%)	- Not specified - Abstract states signs not compatible with conjunctivitis	Day 16.67± 9.29	Full ophthalmological assessment by ophthalmologist
Zhou Y, et al.^(36)^	8/121 (6.6%)	- Itching - Conjunctival hyperemia - Aqueous secretion - Discharge - Foreign body sensation	Not specified	EMR, eye examination with a penlight.
Colavita F, et al.^(37)^	1 (case report)	- Conjunctival hyperemia - Chemosis - Epiphora	Day 2 to 21	Full ophthalmological assessment by ophthalmologist
Chen Lu, et al.^(38)^	1 (case report)	- Bilateral conjunctival injection Watery discharge Conjunctival follicles - Tender & palpable preauricular lymph nodes	Day 13	Full ophthalmological assessment by ophthalmologist
Cheema M, etal.^(39)^	1 (case report)	- Unilateral keratoconjunctivitis Swollen eyelid Conjunctival follicles Aqueous/mucous discharge Subepithelial infiltrates - Pseudodendrite - Tender and palpable preauricular lymph nodes	Day 1	Full ophthalmological assessment by ophthalmologist
Li X, et al.^(40)^	2 (case report)	- Conjunctival hyperemia - Viscous discharge	Day 3	Full ophthalmological assessment by ophthalmologist
Sarma P, et al.^(28)^	3.17% (6 studies, 854 patients)	- Conjunctivitis/red eye	Pooled analysis	Pooled analysis of 6 studies Conjunctivits & red eye pooled in same category

EMR= Electronic medical records.

Overall, ocular manifestations are not common in COVID-19 patients. Six of the case series show less than 5% of patients with any sign, while two report no manifestations. Wu et al. report on hospitalized patients with moderate to severe pneumonia; their findings show 31.6% of patients with ocular signs. They also report that according to univariate analysis, patients with ocular symptoms were more likely to suffer more severe presentations of the disease.

The timing of ocular manifestations during the evolution of COVID-19 is ill-defined. Six studies report ocular findings in eight patients before day 5 of the disease. A case report describes conjunctival congestion more prominently from day 8^(38)^. Another study reports on a patient who showed ocular symptoms from day 8 to 12, characterized by congestion and tearing^(33)^. An additional series describes manifestations averaging from day 7 until day 25^(35)^. Two studies do not specify the onset of manifestations, while the remaining report no ocular findings.

Sarma et al also conducted a meta-analysis of pooled patients across 6 studies (5 items included in the current revision; the other one was discarded during the screening process) to study ocular manifes tations. They concluded the proportion of patients presenting with conjunctivitis/red eye was 3.17% (95% C.I. 1.16 to 6.13)^(28)^.

#### Recommendations to prevent propagation of the virus

Nine articles describe measures that can be implemented in ophthalmology departments and practices in order to prevent SARS-CoV-2 infection. Three are literature reviews^(25-27)^ and six are intervention protocols with low levels of evidence^(41-46)^.

Throughout the four intervention descriptions by Chinese authors, recommendations are based on general strategies built upon a three-level hierarchy system employed in mainland China and Hong Kong: administrative control measures, environmental control measures and the use of personal protective equipment (Table 5).

**Table 5:** Current recommendations on the management of eye care services during the COVID-19 epidemic

Hierarchy	Recommendations
Administrative Control	- Patient workflow management, including rescheduling and reducing non-urgent appointments^(25-27,41,42,44-46)^ - Patient triage algorithms^(25-27,41,42,44-46)^ - TOCC questionnaires(^25,27,41,45,46^’ - Temperature checks^(25,41,45,46)^ - Reducing aerosol-inducing procedures (avoid non-contact air-puff tonometer)^(25,27,41,42,45)^ - Tonopen is recommended^(25,41,45,46)^ - Avoiding accompanying persons if possible^(25,45,46)^ - Minimizing examination time and extending waiting time between examinations^(25,42,45,46)^ - Requesting diagnostic aids only if critical to decision making^(25,45,46)^ - Rescheduling surgical cases according to level of urgency^(25,41,43,45,46)^ - Promote the use of tele-assistance/tele-medicine to orient patients about their condition^(25,27,42,44-46)^ - Using indirect ophthalmoscopy for fundus evaluation^(25,27,41,43,45,46)^ - Adapting waiting rooms to maintain two-meter space between patients^(25,27,41,45,46)^ - Specific recommendations are made for each subspecialty, according to three levels of care: emergency, urgent and routine^(45)^ - Reduce admission time and avoid paperwork exchange^(45,46)^ - Requesting specific informed consent to authorize care in the context of the pandemic^(45,46)^ - In the case of a COVID-19 positive patient, ophthalmological care should be provided in a multi-specialty hospital^(27,45,46)^ - Patients with conjunctivitis to be evaluated in an isolated office with maximum protection^(45)^ - Patients with conjunctivitis or keratoconjunctivitis should be asked about risk factors for COV1D-19^(25)^ - Choose the shortest procedure and local anesthesia over general anesthesia^(27,45,46)^ - Perform chest x-rays on all patients requiring surgery^(45)^
Environmental control	- Use of ventilation and HEPA units^(27,41,45)^ - No-AC policy if possible^(45)^ - Protective shields on slit lamps^(25,27,41,43,45,46)^ - Avoid talking during the evaluation^(27,45,46)^ - Disinfection guidelines for office spaces and equipment^(25,27,41,43,45,46)^ - Ultraviolet light sterilization units can be installed^(45)^
Use of personal protective equipment (PPE)	- Universal masking:^(41,45,46)^ - Use of surgical masks^(25,27,43,46)^ (in non-suspect patients) - N95 respirators for health staff: in suspected COVID-19 cases;^(41,46)^ and aerosol-generating procedures^(25)^ - Eye protection (goggles)^(25,27,41,43,45,46)^ - Full personal protective equipment in suspicious and confirmed cases^(25,27,41,45,46)^ - Long-sleeved gowns (non-sterile and waterproof) if COVID positive or suspected patients are to be treated^(25,45,46)^ - Use of long gloves^(25,27,45,46)^ - The patient should wear gloves during the evaluation^(41)^ - Hand washing with soap and water and/or alcohol gel^(25^'^27,41,43,45,46)^ - Testing for symptomatic staff members or self-isolation for 7 days^(25,27,46)^ - Avoid touching eyes, nose, and mouth^(26,43,46)^ - Prophylactic use of hydroxychloroquine under internal medicine supervision^(45)^

The review by Lyndon et al. gives evidence for contact-lens practices, concluding that there is no evidence suggesting contact lens wearers who are asymptomatic should cease using contact lenses due to an increased risk of developing COVID-19. It also states there is no evidence suggesting that wearing prescription glasses provides protection against SARS-CoV-2^(26)^.

#### References in the analyzed literature

Among the screened records, 47% referenced the case series by Xia et al.^(5)^ in which 1 of 30 patients had positive RT-PCR results in conjunctival secretions (1.3%); this was the most cited item. Similarly, 40% referenced a letter by Lu et al. stating the hypothesis of transmission through the conjunctiva^(47)^. The rest of the screened documents were referenced at an average rate of 3%.

## DISCUSSION

We found that current recommendations regarding COVID-19 and ophthalmology are based on levels of evidence 4 and 5 according to the Oxford CBME methodology grading system. The amount of research conducted to date is limited; the nature of the disease and the scarcity of cases with ocular involvement pose challenging circumstances for research efforts. As the pandemic evolves so will the need for further data to bolster our understanding on COVID-19, its implications for eye care and the outcomes of the implemented strategies.

In a small percentage of patients, SARS-CoV-2 RNA has been isolated in the tear film. In the published literature, positive findings were reported in 0 to 7.14% of subjects across different studies; Sarma et al. found that the virus was present in 1.95% (95% C.I. 0.74 to 4.11) of samples^(28)^. Possible explanations for a negative finding of viral particles on the ocular surface have been set forth: sensitivity of the tests; time of sampling collection; antimicrobial mechanisms of the conjunctiva; collection techniques; and washing of viral particles by tearing and passage to the nasopharynx through the lacrimal duct^(6,48)^.

The reviewed publications showed wide heterogeneity in their methodology. First, the time of sample collection, counted from the onset of any symptom, varied on average from 2 to 18 days, and in some studies was not even specified. Second, the number of samples taken was not consistent across the studies and even within them; some, such as case reports, performed only one test, and others up to four. Lastly, the methods for sample collection and processing included conjunctival swabbing and RT-PCR in the majority of the studies, while one collected tears using Schirmer strips and two also performed viral isolation through cell culture. Therefore, pooling of the data was not deemed appropriate. Until further evidence is available, we cannot rule out the possibility of viral particles being present in tears and conjunctiva; therefore, precautionary measures should be insisted upon.

Possible conjunctival transmission is mainly based on anecdotal reports of HCW who did not use eye protection^(40)^, early conjunctival congestion symptoms reported in some patients, or the presence of viral RNA in the tear film^(15,16)^. Several hypothetical transmission me chanisms have been proposed. First, the virus adheres to ACE2 receptors found on the conjunctival and corneal epithelia^(49,50)^. Second, the nasolacrimal duct would serve as a pathway from the conjunctiva to the upper respiratory tract where the virus can infect the host^(51)^. However, some authors believe that this is not enough evidence to confirm the conjunctival route of transmission and suggest the following counter-arguments: a) the presence of the ACE2 protein in the conjunctiva is low compared to the lung; b) lactoferrin and secretory IgA present in the tears could eliminate the virus; and c) the presence of the virus in tears could be explained by fomites transmitted to the conjunctiva via splashed droplets or by direct contact with a contaminated hand^(51)^. Qiao et al. report that the overall incidence of COVID-19 among eye professionals across 10 hospitals was 2.52% (95% CI: 1.68-3.63%); the incidence of the disease was similar in ophthalmologists as that of general practitioners^(52)^. Although there is currently no confirmed conjunctival transmission route, the authors agree with the WHO and other organizations´ recommendations that PPE must include eye protection such as goggles or face shields^(2,24)^.

A wide range of non-specific ocular manifestations has been reported for COVID-19; at this time, a characteristic presentation has not been clearly determined. The most common ocular manifestations were conjunctival hyperemia and watery discharge. This presentation varies, as can be observed with the report of Cheema et al. on a patient presenting with keratoconjunctivitis^(39)^. The methodology of the reports also varies widely, as can be seen in the approaches taken by two of the studies. Wu et al. examined hospitalized COVID-19 patients who were more severely ill, including intubated patients, finding that patients with ocular manifestations presented more severe systemic disease or abnormal findings on blood tests^(16)^. Chen et al. obtained data via telephone, face-to-face surveys, or a smartphone application^(33)^. If statistical measures to overcome the underlying studies limitations and the appropriateness of a pooled analysis are accepted, then Sarma et al. report that conjunctivitis/red eye is featured in 3.17% (95%CI 1.16 to 6.13) of patients^(28)^. The normal prevalence of dry eye and allergic conjunctivitis could explain some of the reported ocular symptoms; other explanations may be related to poor hygiene or face mask misuse. Overall, severe eye manifestations have not been reported, and more specific observations will be needed to establish a particular set of findings.

Because of a lack of interventional studies, recommendations in current publications are based on lower-level evidence. Most derive from the experience gained and the general strategies implemented during the SARS outbreak in 2002-2004^(41,53)^. Administrative, environmental and PPE measures should be implemented, and attention should be given to protecting both HCW and patients. There is a need for studies that test or certify the effectiveness of these intervention measures during the current SARS-CoV-2 pandemic; however, erring on the side of safety is currently necessary. As better evidence continues to accumulate, it will be important to update these measures and adapt to a rapidly evolving scenario.

It was of particular interest to the authors that a considerable proportion of the analyzed literature referenced an anecdotal report about a Chinese respiratory expert who contracted COVID-19 despite having worn appropriate PPE except eye protection^(2,47,54)^. This report was cited in 40% of the screened publications and might have been the initial source for many of the recommendations on ocular protection^(47)^. This phenomenon illustrates how quickly information with low levels of evidence can be widely disseminated.

This is the first review on the level of evidence for ophthalmology recommendations and COVID-19 conducted to date. It includes an independent full text review performed by five reviewers. This study was limited to SARS-CoV-2; therefore, data obtained from previous coronavirus epidemics might have been outside of its purview. Some of that information may be as important as what has been reviewed here; the article by Seah et al. on the evidence of ocular involvement in coronavirus cases might have served to counteract this shortcoming. An additional limitation is the dependence on third-party translation for Chinese publications; in order to address this limitation, translations were performed on multiple platforms, and an additional reviewer was added to these papers. Publication bias was approached by searching through grey literature, editorials, opinion pieces, pre-published works, and non-peer-reviewed articles alongside traditional publications, without language restrictions.

COVID-19 is a novel disease that has caused a pandemic unlike any we have experienced in modern medicine, not only because of the characteristics of the disease, but because of the speed of the spread of information. The pandemic has only begun to be studied properly; therefore, the scarcity of medical appraisals, randomized controlled trials and case control studies is not surprising. In this structured review we classified the available evidence and recommendations relating to ophthalmology during the COVID-19 pandemic.

Overall, the level of available evidence for current recommendations is rising.

Currently, there is not sufficient evidence to rule out the possibility of viral particles being present in tears and conjunctiva. A few hypothetical mechanisms have been proposed suggesting that the conjunctiva may act as an entry route for SARS-CoV-2. With the varying degrees of evidence supporting or refuting it, conjunctival transmission remains controversial. Ocular manifestations are not common in COVID-19 patients; evidence suggests that they can resemble a viral infection of the ocular surface, with hyperemia and watery discharge as the cardinal signs. Most of the literature published to date consists of anecdotal reports, editorials, and opinion pieces with a high level of cross-referencing. The documents that currently contribute to expanding the knowledge of ocular involvement in COVID-19 are ranked as having low levels of evidence. Most recommendations are based on the strategies implemented in Asian countries during previous CoVs outbreaks, many of them are likely to prevail and set new standards of preventive measures in health systems. During the evolution of this worldwide phenomenon, reliable information will be crucial to elucidate recommendations to mitigate the propagation of SARS-CoV-2. As new studies and cases are reported, it will be fundamental to evaluate their level of evidence to correctly assess the recommendations and adapt them to the local circumstances.
